# Method Comparison of Three Automated D-Dimer Assays

**DOI:** 10.3390/diagnostics16193119

**Published:** 2026-09-25

**Authors:** Bağnu Dündar, Betül Özbek Kurtul, Beyzanur Badilli, Sevgi Koçyiğit Sevinç, Elif Ertaş, Asiye Gök Yurttaş

**Affiliations:** 1 Department of Medical Biochemistry, Faculty of Medicine, Atlas University, 34408 Istanbul, Türkiye; bagnu.dundar@atlas.edu.tr (B.D.); betul.ozbek@atlas.edu.tr (B.Ö.K.); 2 Department of Chemistry, Yildiz Technical University, 34220 Istanbul, Türkiye; beyzanurbadilli@gmail.com; 3 Department of Biophysics, Faculty of Medicine, Kütahya Health Sciences University, 43100 Kütahya, Türkiye; sevgi.kocyigitsevinc@ksbu.edu.tr; 4 Department of Biostatistics and Medical Informatics, Faculty of Medicine, Istanbul Atlas University, 34408 Istanbul, Türkiye; elif.ertas@atlas.edu.tr

**Keywords:** D-dimer, method comparison, analytical agreement, Passing–Bablok regression, Bland–Altman analysis, venous thromboembolism, coagulation analyzer, intermethod variability

## Abstract

**Background and Objective:** D-dimer results differ across analytical platforms, which may affect their clinical interpretation. This study aimed to compare the analytical agreement and threshold-based classification of three routinely used D-dimer analyzers. **Methods:** Of 150 consecutive residual citrated plasma specimens, 81 patient specimens with valid results on the Sysmex CS-2500, Tokra Novae MTI, and VIDAS 3 analyzers were included. Agreement was assessed using the intraclass correlation coefficient, Passing–Bablok regression, Bland–Altman analysis, and classification concordance at the fixed (500 ng/mL FEU) and age-adjusted thresholds. VIDAS 3 served solely as the comparator method. **Results:** Overall agreement was moderate to good; however, the platforms were not fully interchangeable. Sysmex CS-2500 and VIDAS 3 showed the closest agreement, with no significant constant or proportional bias, whereas Tokra Novae MTI exhibited significant proportional bias. Mixed classification across the three analyzers occurred in 21/81 patients (25.9%) at the fixed threshold and in 18/81 patients (22.2%) at the age-adjusted thresholds. **Conclusions:** Clinically relevant inter-platform differences persisted despite acceptable average agreement. Therefore, D-dimer results should not be regarded as interchangeable across analyzers, particularly near clinical decision thresholds, and platform-specific verification remains essential.

## 1. Introduction

Venous thromboembolism (VTE), which comprises deep vein thrombosis and pulmonary embolism, remains a major cause of morbidity and mortality worldwide; therefore, its timely and accurate diagnosis is essential to avoid both under- and overtreatment [1,2]. D-dimer, a soluble degradation product of cross-linked fibrin, is an integral component of the diagnostic algorithm for suspected VTE, mainly because of its high negative predictive value in patients with a low or intermediate pretest probability [1,2,3]. Its central role in clinical decision-making is supported by major international guidelines, including those of the European Society of Cardiology and the American Society of Hematology, which recommend D-dimer testing as a first-line investigation to safely exclude VTE without further imaging in appropriately selected patients [1,4,5].

However, the analytical behavior of this marker is constrained by the nature of the analyte itself. During clot formation, thrombin-mediated fibrin polymerization is followed by ε-(γ-glutamyl)lysine cross-linking between the γ-chains of adjacent monomers, a reaction catalyzed by activated factor XIII (FXIIIa) [6,7,8]. Plasmin-mediated degradation of this covalently stabilized network releases fragments in which two D domains remain linked through the FXIIIa-generated γ–γ cross-link; this entity is referred to as D-dimer [9,10]. Because this epitope arises only from cross-linked fibrin, it is a specific indicator of coagulation followed by fibrinolysis [9,11]. Importantly, however, the circulating analyte is not a single molecular species. Plasma contains a heterogeneous spectrum of high-molecular-weight derivatives, ranging from the minimal DD unit to DD/E complexes and larger species exceeding 1 MDa; consequently, no single fragment can serve as a universal calibrator [9,10,11].

This molecular heterogeneity is the underlying source of the methodological variability observed in clinical practice. Commercially available immunoassays use different monoclonal antibody clones that target distinct neoepitopes within this fragment population, differ in their detection principles and calibration materials, and may report results in either D-dimer units or fibrinogen-equivalent units (FEU) [12,13,14,15]. In general, the platforms in routine use include latex-enhanced immunoturbidimetric assays integrated into general coagulation analyzers and enzyme-linked fluorescence assays (ELFA) performed on dedicated immunoassay systems; both assay types have shown acceptable stand-alone analytical performance in previous validation studies [16,17,18,19]. Nevertheless, no internationally harmonized reference measurement procedure or calibration standard is currently available, a limitation explicitly recognized by the International Federation of Clinical Chemistry and Laboratory Medicine and by the Scientific and Standardization Committee on Fibrinolysis of the International Society on Thrombosis and Haemostasis [12,20]. Although efforts to establish a candidate World Health Organization international standard represent progress, harmonization has not yet been achieved in routine practice [12]. Consequently, D-dimer values obtained on different analyzers are not necessarily numerically interchangeable, even when each platform performs within its own validated range [12,17,21].

The clinical implications of this lack of interchangeability are tangible. D-dimer is used predominantly as a binary rule-out test against a decision threshold, most commonly 500 ng/mL FEU, although assay-specific thresholds should, in principle, be applied [22]. If two platforms show constant or proportional bias relative to each other, a patient classified as negative by one analyzer may be classified as positive by another, with consequences ranging from unnecessary imaging and anticoagulation to a missed diagnosis of thromboembolic disease [16,23]. This vulnerability is greatest in the immediate vicinity of the threshold, where small analytical differences translate directly into different management decisions [12,17]. The problem is further compounded by two developments: the increasing adoption of age-adjusted thresholds (age × 10 ng/mL FEU in patients aged ≥50 years), which reduce false-positive results without compromising sensitivity [24,25], and the growing use of absolute D-dimer concentrations as semi-quantitative markers of disease severity or prognosis in conditions such as disseminated intravascular coagulation and COVID-19-associated coagulopathy [21,26]. Both applications presuppose a degree of numerical comparability between platforms that has not been formally demonstrated.

Previous method-comparison studies have largely been limited to pairwise comparisons on the continuous measurement scale, and few have examined whether agreement in measured concentrations is preserved at the level of clinical classification, particularly when age-adjusted rather than fixed thresholds are applied. This distinction is not trivial, because concordance at one level does not necessarily imply concordance at the other [17,18], as has also been shown for other coagulation parameters compared across laboratories [27]. Accordingly, the present study evaluated the analytical performance and intermethod comparability of D-dimer measurements obtained on three platforms—the Sysmex CS-2500 and the Tokra Novae MTI, both of which use latex-enhanced immunoturbidimetry, and the VIDAS 3, which uses ELFA—in a cohort of patients with valid results on all three analyzers. We assessed overall absolute agreement, pairwise constant and proportional bias, individual-level agreement, and clinical classification concordance at both fixed and age-adjusted cutoffs, with the VIDAS 3 serving as the comparator [28,29]. We hypothesized that overall agreement would be acceptable, but that the two immunoturbidimetric platforms would show systematic proportional bias relative to ELFA, resulting in discordant classification in a clinically non-negligible proportion of patients with values close to the decision threshold, and that this discordance would be greater with age-adjusted than with fixed cutoffs.

## 2. Materials and Methods

### 2.1. Study Design and Setting

This prospective method-comparison study was conducted at the Central Laboratory of Istanbul Atlas University Hospital. Its primary objective was to evaluate the analytical agreement, systematic bias, and clinical classification concordance of D-dimer measurements obtained simultaneously on three different analytical platforms.

### 2.2. Sample Collection

A total of 150 residual citrated plasma samples were prospectively collected after approval had been obtained from the Institutional Review Board/Ethics Committee on 22 June 2026. Samples were obtained from consecutive patients for whom D-dimer testing had been requested as part of routine clinical care. Only residual specimens remaining after all requested laboratory analyses had been completed and the final clinical reports had been released were eligible for inclusion. No additional blood samples were drawn, and no additional procedures were performed for research purposes. All specimens were de-identified before analysis. To reflect routine laboratory practice, samples were selected irrespective of patient diagnosis, age, sex, or clinical department. Eligible residual specimens were randomly selected from consecutive laboratory submissions during the predefined study period.

The sample flow and unit of analysis were defined as follows. The initial pool comprised 150 residual citrated plasma specimens obtained from consecutive routine D-dimer requests. For the comparative analysis, only specimens that met all predefined inclusion criteria and yielded a valid D-dimer result on each of the three analyzers were retained as complete cases. The final complete-case dataset contained one eligible specimen per patient; thus, the 81 analytical observations corresponded to 81 individual patients, and repeat specimens were not treated as independent observations. The remaining 69 specimens were excluded because they did not meet one or more of the predefined preanalytical, sample-adequacy, or result-completeness requirements, namely insufficient residual volume, unacceptable specimen quality (e.g., hemolysis, lipemia, icterus, clotting, or improper collection), an interval of 2 h or longer before analysis, or the absence of a valid result from at least one analyzer. Therefore, the study population for statistical comparison was defined by the availability of complete valid results on all three platforms rather than by selective inclusion based on clinical or demographic characteristics.

In the final analytical cohort of 81 patients, age and sex were recorded because age was required for the age-adjusted threshold analysis and sex was used to describe the study population. Detailed diagnoses, clinical pretest probability scores, imaging findings, and the specific clinical indication for each D-dimer request were not collected in the de-identified method-comparison dataset. Therefore, the study was designed to evaluate analytical comparability under routine laboratory conditions rather than diagnostic performance in specific clinical subgroups.

Inclusion Criteria

Samples that met all of the following criteria were included:

- Citrated plasma specimens collected according to standard venipuncture procedures;
- Sufficient residual sample volume for simultaneous analysis on all three analytical platforms;
- Availability of the specimen for measurement on all analyzers under identical preanalytical conditions;
- A time interval of no more than 2 h between specimen collection and analysis.

Exclusion Criteria

Samples were excluded if any of the following conditions were present:

- Visible hemolysis, lipemia, or icterus;
- Clotted or improperly collected specimens;
- Insufficient sample volume;
- Specimens kept for ≥2 h between collection and analysis.

### 2.3. Analytical Procedures

D-dimer concentrations were measured simultaneously on three automated analytical systems:

- Sysmex CS-2500 (Sysmex Corporation, Kobe, Japan), using the INNOVANCE D-Dimer latex-enhanced immunoturbidimetric assay;
- Tokra Novae MTI (Tokra Medical Technology, Ankara, Türkiye), using the manufacturer-configured latex-enhanced immunoturbidimetric D-dimer reagent system;
- VIDAS 3 (bioMérieux, Marcy-l’Étoile, France), using the VIDAS D-Dimer Exclusion II assay, a two-step enzyme-linked fluorescence assay (ELFA).

Residual citrated plasma was analyzed after routine clinical testing had been completed. Only specimens with sufficient residual volume and acceptable preanalytical quality were used, and all three measurements were completed within 2 h of specimen collection. No additional blood was drawn for research purposes. Whenever the volume permitted, the same residual plasma aliquot was analyzed on all three platforms to minimize between-platform preanalytical differences. Measurements were performed according to the manufacturers’ current instructions for use, including platform-specific calibration and quality-control procedures.

All analyses were performed in accordance with the manufacturers’ instructions and routine laboratory operating procedures. To minimize preanalytical variability, all measurements were performed under identical specimen-handling conditions and without unnecessary delay between analyses. Because analytical platforms may report D-dimer concentrations in different units and with different calibration schemes, all results were converted to a common reporting unit (fibrinogen-equivalent units, FEU) before statistical comparison. The analytical performance and method-comparison procedures were designed in accordance with internationally accepted recommendations and standards, including CLSI EP09-A3 (Measurement Procedure Comparison and Bias Estimation Using Patient Samples), CLSI EP05-A3 (Evaluation of Precision of Quantitative Measurement Procedures), the requirements of ISO 15189:2022 (Medical laboratories—Requirements for quality and competence) [30], the recommendations of the International Federation of Clinical Chemistry and Laboratory Medicine (IFCC), and the guidance of the International Society on Thrombosis and Haemostasis (ISTH) on D-dimer reporting and harmonization.

During the study period, all three analytical platforms (Sysmex CS-2500, Tokra Novae MTI, and VIDAS 3) were in routine clinical use for D-dimer measurement in the Central Laboratory. Thus, the three systems were not research-only instruments but part of the laboratory’s daily diagnostic workflow. For this method-comparison study, each eligible residual specimen was measured on all three platforms under the same preanalytical conditions, and no single analyzer was designated as the laboratory’s exclusive routine method or as a gold-standard reference method. The investigation was designed to compare analytical agreement and clinical classification among the three routinely used systems, not their workflow or health-economic performance. Accordingly, turnaround time, reagent and consumable costs, hands-on time, maintenance burden, throughput, and ease of use were neither prospectively recorded nor formally compared, and the present data do not allow the platforms to be ranked according to these operational criteria.

### 2.4. Statistical Analysis

Statistical analyses were performed using IBM SPSS Statistics for Windows, Version 25.0 (IBM Corp., Armonk, NY, USA) and MedCalc Statistical Software, Version 23.6.1 (MedCalc Software Ltd., Ostend, Belgium). A total of 81 patients with valid D-dimer results on all three analyzers (Sysmex CS-2500, Tokra Novae MTI, and VIDAS 3) were included, and all analyses were conducted in this same complete-case population.

Demographic characteristics were summarized using descriptive statistics. Age was expressed as the mean, standard deviation, median, first and third quartiles, and minimum–maximum values, whereas sex was expressed as frequency and percentage.

Overall absolute agreement among the three analyzers was assessed using the intraclass correlation coefficient based on a two-way mixed-effects, absolute-agreement, single-measurement model [ICC(A,1)]. The ICC estimate was reported together with its 95% confidence interval. Constant and proportional bias between analyzer pairs were evaluated by Passing–Bablok regression analysis [28] on the original D-dimer measurement scale. Constant bias was considered present when the 95% confidence interval of the intercept did not include 0, and proportional bias was considered present when the 95% confidence interval of the slope did not include 1.

Because the D-dimer measurements spanned a wide range, showed a markedly right-skewed distribution, and exhibited concentration-dependent differences between methods, logarithmic transformation was applied only in the Bland–Altman analysis [29]. The Bland–Altman results were back-transformed and reported as geometric mean ratios with 95% ratio limits of agreement. The ICC, Passing–Bablok regression, and all threshold-based classification analyses were performed on the original D-dimer values.

Because this was a method-comparison study rather than a conventional comparison of group means, formal normality testing of the raw D-dimer measurements was not used as a prerequisite for selecting the principal statistical methods. Passing–Bablok regression was chosen because it is specifically designed for the comparison of measurement procedures and does not require normally distributed data. For the Bland–Altman analysis, the wide concentration range, marked right skewness, and concentration-dependent variability were considered the more relevant distributional features; therefore, logarithmic transformation was used to stabilize the measurement scale and to express agreement in terms of ratios.

For descriptive interpretation, ICC values were categorized as poor (<0.50), moderate (0.50–0.75), good (0.75–0.90), or excellent (>0.90) [31]. Cohen’s kappa values were interpreted as indicating slight (0.01–0.20), fair (0.21–0.40), moderate (0.41–0.60), substantial (0.61–0.80), or almost perfect (0.81–1.00) agreement [32]. Ninety-five percent confidence intervals were reported for the principal agreement and classification estimates. Confidence intervals for geometric mean ratios were calculated on the logarithmic scale and then back-transformed; Wilson score intervals were used for proportions, including overall agreement and relative classification measures; and asymptotic 95% confidence intervals based on the standard error were reported for Cohen’s kappa.

Agreement in negative/positive classification across analyzers was evaluated using two cutoff approaches. In the fixed-cutoff analysis, a threshold of 500 ng/mL FEU was applied. In the age-adjusted analysis, the cutoff was defined as 500 ng/mL FEU for patients aged <50 years and as age × 10 ng/mL FEU for patients aged ≥50 years [21]. Pairwise classification agreement was assessed using the overall percentage agreement and Cohen’s kappa coefficient.

Passing–Bablok results were additionally visualized for each pairwise comparison by plotting the observed patient samples, the fitted Passing–Bablok regression line, and the line of identity (y = x). In addition to the primary logarithmic Bland–Altman analysis, raw-scale and percentage-difference Bland–Altman plots were constructed for Tokra Novae MTI versus Sysmex CS-2500. Raw-scale differences were defined as Tokra − Sysmex, and percentage differences as 100 × (Tokra − Sysmex)/[(Tokra + Sysmex)/2]; mean differences and 95% limits of agreement were calculated as the mean ± 1.96 SD. To illustrate specimen-level variation, the original D-dimer values obtained with all three analyzers were also displayed for each of the 81 patients in a patient-level dot plot. For the threshold-based analyses, each analyzer pair was cross-classified into four mutually exclusive categories: both below the threshold; both at or above the threshold; analyzer A below and analyzer B at or above the threshold; and analyzer A at or above and analyzer B below the threshold.

Because no universally accepted reference method exists for D-dimer measurement, VIDAS 3 was used solely as a comparator method. Relative sensitivity, specificity, positive predictive value, and negative predictive value were calculated for Sysmex and Tokra on the basis of the VIDAS classification. These estimates were interpreted as measures of relative classification performance and not as measures of true diagnostic accuracy against confirmed pulmonary embolism. All tests were two-sided, and a *p*-value of <0.05 was considered statistically significant.

## 3. Results

A total of 81 patients with valid D-dimer results on all three analyzers (Sysmex CS-2500, Tokra Novae MTI, and VIDAS 3) were included in the analysis. The mean age of the study population was 42.4 ± 20.0 years (median, 40.0 years; interquartile range, 26.0–56.0 years), and ages ranged from 3 to 90 years. The cohort was predominantly female (62 patients, 76.5%), and the remaining 19 patients (23.5%) were male.

### 3.1. Overall Agreement Among the Three Analyzers

The single-measurement, absolute-agreement intraclass correlation coefficient [ICC(A,1)] across the three analyzers was 0.739 (95% CI: 0.695–0.766). According to the predefined interpretation criteria, the point estimate fell within the moderate range, whereas the 95% confidence interval extended from the moderate into the good range (Table 1). Although this indicates an acceptable level of concordance among the three analyzers, the ICC remained below the level generally required for full interchangeability at the individual-measurement level.

### 3.2. Passing–Bablok Regression Analysis

Pairwise comparisons using Passing–Bablok regression are summarized in Table 2 and illustrated in Figure 1, Figure 2 and Figure 3. For the Sysmex–Tokra comparison, the 95% CI of the intercept included zero (8.70; 95% CI: −30.48 to 49.97), whereas the 95% CI of the slope excluded 1 (0.545; 95% CI: 0.450–0.645), indicating the absence of significant constant bias but the presence of significant proportional bias. The slope of 0.545 indicates that Tokra values became progressively lower than Sysmex values as D-dimer concentrations increased.

For the Sysmex–VIDAS comparison, the 95% CIs of the intercept (3.79; 95% CI: −37.46 to 39.92) and the slope (1.011; 95% CI: 0.892–1.135) included 0 and 1, respectively, indicating no significant constant or proportional bias between these two analyzers.

For the Tokra–VIDAS comparison, no significant constant bias was detected (intercept, 22.56; 95% CI: −83.85 to 97.78); however, significant proportional bias was present, because the confidence interval of the slope (1.624; 95% CI: 1.236–2.188) excluded 1. Overall, the best analytical agreement was observed between Sysmex and VIDAS, whereas Tokra showed proportional bias relative to both of the other analyzers, suggesting that clinically meaningful numerical discrepancies may arise at higher D-dimer concentrations.

Passing–Bablok regression plots are presented in Figure 1, Figure 2 and Figure 3. The Sysmex–Tokra regression line deviated increasingly from the identity line at higher D-dimer concentrations, visually supporting the proportional bias identified in Table 2 (slope, 0.545; 95% CI: 0.450–0.645; Figure 1). In contrast, the Sysmex–VIDAS regression line remained close to the identity line, consistent with the absence of significant constant or proportional bias (Figure 2). The Tokra–VIDAS plot likewise showed increasing divergence from the identity line, consistent with significant proportional bias (slope, 1.624; 95% CI: 1.236–2.188; Figure 3).

### 3.3. Bland–Altman Agreement Analysis

The logarithmically transformed Bland–Altman analysis yielded a geometric mean ratio of 0.652 for Tokra/Sysmex, indicating that Tokra values were, on average, approximately 65.2% of Sysmex values (Table 3). The VIDAS/Sysmex ratio was close to unity (1.021), reflecting comparable mean measurement levels for these two analyzers. The VIDAS/Tokra ratio was 1.566, indicating that VIDAS values were, on average, approximately 56.6% higher than Tokra values. Notably, the 95% limits of agreement were wide for all three analyzer pairs (Figure 4, Figure 5 and Figure 6), indicating that, despite acceptable mean-level agreement in some comparisons, considerable differences may occur in individual patient samples.

Additional Tokra–Sysmex Bland–Altman plots on the original measurement scale are presented in Figure 7 and Figure 8. The mean raw-scale difference (Tokra − Sysmex) was −270.9 ng/mL FEU, with 95% limits of agreement ranging from −1653.1 to 1111.4 ng/mL FEU (Figure 7). The mean percentage difference was −39.4%, with 95% limits of agreement ranging from −164.4% to 85.6% (Figure 8), indicating that Tokra measurements were lower on average and showed substantial dispersion across individual samples. Patient-level measurements obtained with all three methods are displayed in Figure 9. The median within-patient absolute range was 159.76 ng/mL FEU (IQR: 99.40–203.09) when all three analyzers yielded values below 500 ng/mL FEU, compared with 1476.68 ng/mL FEU (IQR: 946.30–2165.34) when all three yielded values at or above 500 ng/mL FEU, indicating that the largest absolute numerical dispersion occurred predominantly at higher D-dimer concentrations.

### 3.4. Classification Agreement at the Fixed Cutoff

Detailed pairwise cross-classification at the conventional fixed cutoff of 500 ng/mL FEU is shown in Table 4. For Sysmex–Tokra, 51 patients (63.0%) had values below 500 ng/mL FEU on both analyzers and 16 (19.8%) had values at or above 500 ng/mL FEU on both; 14 patients (17.3%) were discordantly classified, resulting in an overall agreement of 82.7% (κ = 0.576). For Sysmex–VIDAS, 50 patients (61.7%) were concordantly below the threshold and 21 (25.9%) were concordantly at or above it, with 10 discordant classifications (12.3%); this pair showed the highest agreement at the fixed cutoff (87.7%, κ = 0.717). For Tokra–VIDAS, 48 patients (59.3%) were below the threshold on both analyzers and 15 (18.5%) were at or above it on both, whereas 18 patients (22.2%) were discordantly classified. Notably, 12 patients (14.8%) had values below 500 ng/mL FEU on Tokra but at or above 500 ng/mL FEU on VIDAS, compared with 6 patients (7.4%) who showed the opposite pattern. Across all three analyzers, 46/81 patients (56.8%) had values below 500 ng/mL FEU with all methods, 14/81 (17.3%) had values at or above 500 ng/mL FEU with all methods, and 21/81 (25.9%) showed a mixed classification pattern. According to the predefined kappa criteria, agreement was moderate for Sysmex–Tokra and Tokra–VIDAS and substantial for Sysmex–VIDAS; the 95% confidence intervals for overall agreement and kappa are provided in Table 4.

### 3.5. Classification Agreement at the Age-Adjusted Cutoff

Detailed pairwise cross-classification using the age-adjusted cutoff (500 ng/mL FEU for patients aged <50 years; age × 10 ng/mL FEU for patients aged ≥50 years) is shown in Table 5. For Sysmex–Tokra, 53 patients (65.4%) had values below the patient-specific threshold on both analyzers and 14 (17.3%) had values at or above it on both, whereas 14 patients (17.3%) were discordantly classified (82.7% agreement, κ = 0.550). For Sysmex–VIDAS, 56 patients (69.1%) were concordantly below the age-adjusted threshold and 19 (23.5%) were concordantly at or above it; only 6 patients (7.4%) were discordantly classified, resulting in the highest agreement (92.6%, κ = 0.813). For Tokra–VIDAS, 52 patients (64.2%) were below the threshold on both analyzers and 13 (16.0%) were at or above it on both, with 16 discordant classifications (19.8%). Nine patients (11.1%) had values below the age-adjusted threshold on Tokra but at or above it on VIDAS, whereas 7 (8.6%) showed the opposite pattern. Across all three analyzers, 51/81 patients (63.0%) had values below the age-adjusted threshold with all methods, 12/81 (14.8%) had values at or above the threshold with all methods, and 18/81 (22.2%) showed mixed classification. According to the predefined kappa criteria, Sysmex–Tokra and Tokra–VIDAS showed moderate agreement, whereas Sysmex–VIDAS showed almost perfect agreement; the corresponding 95% confidence intervals are reported in Table 5.

### 3.6. Relative Classification Performance Using VIDAS as the Comparator

With VIDAS 3 used strictly as the comparator method, relative classification measures were calculated for Sysmex and Tokra at both cutoffs (Table 6 and Table 7). At the fixed cutoff, Sysmex showed a relative sensitivity of 77.8%, a relative specificity of 92.6%, a relative positive predictive value of 84.0%, and a relative negative predictive value of 89.3%, whereas the corresponding values for Tokra were 55.6%, 88.9%, 71.4%, and 80.0%. These measures quantify classification concordance with VIDAS 3 and should not be interpreted as diagnostic sensitivity, specificity, PPV, or NPV against imaging-confirmed VTE.

At the age-adjusted cutoff, the relative sensitivity, relative specificity, and relative negative predictive value of Sysmex were 86.4%, 94.9%, and 94.9%, respectively, whereas the corresponding estimates for Tokra were 59.1%, 88.1%, and 85.2% (Table 7). These findings indicate closer classification concordance with VIDAS 3 for Sysmex than for Tokra. However, because VIDAS 3 was not a true reference standard and VTE status was not confirmed by imaging, these relative measures cannot establish which analyzer has greater diagnostic accuracy.

## 4. Discussion

In this prospective method-comparison study, we evaluated the analytical agreement, systematic bias, and clinical classification concordance of D-dimer measurements obtained simultaneously on three platforms (Sysmex CS-2500, Tokra Novae MTI, and VIDAS 3) in 81 patients with valid results on all analyzers. Overall agreement was acceptable but did not reach the level generally required for full interchangeability [12,17], and this shortfall was not evenly distributed. Sysmex CS-2500 and VIDAS 3 showed close agreement at both the continuous and classification levels, whereas Tokra Novae MTI exhibited consistent proportional bias relative to both comparators, which translated into clinically relevant classification discordance near the diagnostic threshold [16,23].

The ICC(A,1) of 0.739 (95% CI: 0.695–0.766) had a point estimate within the moderate range, and its confidence interval extended into the good range according to the predefined interpretation criteria [31]. This value remains below the level generally expected for full interchangeability, indicating that D-dimer results from different analyzers should not be treated as numerically equivalent, even when each analyzer performs within its own validated range [12,17,21]. Similar conclusions have been reported for latex-enhanced immunoturbidimetric D-dimer assays verified on different analytical platforms, confirming that comparability cannot be assumed even among assays based on the same detection principle [33]. Passing–Bablok regression clarified the source of this limited agreement. The Sysmex–VIDAS comparison showed neither constant nor proportional bias (slope, 1.011; 95% CI: 0.892–1.135), whereas both the Sysmex–Tokra (slope, 0.545) and Tokra–VIDAS (slope, 1.624) comparisons showed significant proportional bias that became more pronounced at higher D-dimer concentrations. This concentration-dependent divergence is consistent with the molecular heterogeneity of D-dimer, which circulates as a spectrum of fragments of different sizes for which no single calibrator is representative [9,10,11,12]. Consequently, immunoassays based on different antibody clones directed against distinct neoepitopes are inherently prone to platform-specific drift at higher concentrations, a pattern also described in earlier calibration-based comparisons [34]. The Bland–Altman analysis supported this interpretation: the VIDAS/Sysmex geometric mean ratio was close to unity (1.021; 95% CI: 0.953–1.095), whereas Tokra yielded lower concentrations than Sysmex (Tokra/Sysmex, 0.652; 95% CI: 0.556–0.765), and VIDAS yielded higher concentrations than Tokra (VIDAS/Tokra, 1.566; 95% CI: 1.317–1.862). Notably, the 95% limits of agreement were wide for every pair, including Sysmex–VIDAS, indicating that acceptable mean-level agreement does not guarantee comparable results for an individual specimen.

The additional patient-level and original-scale analyses further clarified the concentration-dependent nature and clinical relevance of this disagreement. The median within-patient absolute range across the three platforms was 159.76 ng/mL FEU when all methods yielded values below 500 ng/mL FEU, compared with 1476.68 ng/mL FEU when all methods yielded values at or above 500 ng/mL FEU, indicating that the largest absolute numerical dispersion occurred predominantly at higher D-dimer values. For Tokra versus Sysmex, the raw-scale Bland–Altman mean difference was −270.9 ng/mL FEU and the mean percentage difference was −39.4%, further supporting the tendency of Tokra to yield lower results. Importantly, however, the concentration of large absolute differences at higher values did not preclude clinically relevant discordance across the decision threshold: mixed classification across the three analyzers occurred in 21/81 patients (25.9%) at the fixed 500 ng/mL FEU threshold and in 18/81 patients (22.2%) at the age-adjusted thresholds. Therefore, numerical dispersion at high concentrations and classification discordance around rule-out thresholds should be regarded as distinct but complementary aspects of intermethod comparability.

From a practical perspective, the implications of inter-analyzer disagreement depend on the concentration range. Large absolute differences at clearly elevated D-dimer concentrations may not alter a simple positive/negative classification when all methods yield values above the decision threshold, although they may affect longitudinal monitoring or the interpretation of absolute D-dimer values. In contrast, smaller analytical differences near the fixed or age-adjusted rule-out threshold can determine whether a sample is classified as negative or positive and may therefore influence subsequent imaging decisions. Nevertheless, because standardized clinical pretest probability assessments and imaging-confirmed VTE outcomes were not available in the present study, the observed threshold-crossing discordance should not be interpreted as evidence of missed or delayed VTE diagnoses.

The most clinically important finding was that agreement on the continuous scale did not fully predict agreement at the classification level, and that this relationship depended on the cutoff strategy applied. At the fixed 500 ng/mL FEU threshold, Sysmex–VIDAS agreement was substantial (κ = 0.717), whereas Tokra–VIDAS agreement was only moderate (κ = 0.471). Age adjustment markedly improved Sysmex–VIDAS concordance (92.6%, κ = 0.813) but yielded no comparable benefit for Tokra (κ = 0.486). This asymmetric response indicates that the well-documented benefit of age-adjusted thresholds, namely a reduction in unnecessary imaging without loss of sensitivity [1,4,5,24,25], cannot be assumed to apply uniformly across analyzers. This observation is consistent with previous evidence showing that the performance of age adjustment varies when different D-dimer assays are substituted for one another [35]; that alternative interpretation strategies (age-adjusted versus probability-adjusted) can yield similar negative predictive values but different proportions of negative results depending on the assay used [36]; and that, although age-adjusted cutoffs improve specificity across multiple assays, the magnitude of this benefit differs between platforms [37]. These reports closely mirror the analyzer-dependent pattern observed in the present study. Therefore, an age-adjustment algorithm validated on one platform cannot be assumed to provide an equivalent benefit on a platform with underlying proportional bias.

The relative classification analysis, with VIDAS 3 as the comparator, further illustrates analyzer-dependent differences in classification. Sysmex showed higher relative sensitivity than Tokra at both the fixed and age-adjusted cutoffs, indicating closer agreement with VIDAS 3 for VIDAS-positive classifications. However, these estimates describe agreement with a comparator assay rather than diagnostic performance against confirmed VTE. Because neither imaging-confirmed outcomes nor a true reference measurement procedure was available, the lower relative sensitivity observed for Tokra cannot, by itself, be interpreted as reduced clinical diagnostic sensitivity or as evidence of missed VTE. Rather, these findings support caution when rule-out thresholds or clinical interpretations are transferred between analytical platforms.

With respect to measurement accuracy, the present study cannot establish absolute analytical accuracy or metrological trueness, because no internationally accepted reference measurement procedure or commutable reference material is available for D-dimer. Accordingly, the term accuracy should be understood here in a relative, method-comparison sense. The Passing–Bablok and Bland–Altman results indicated that Sysmex CS-2500 and VIDAS 3 had the closest relative agreement, with no significant constant or proportional bias and a geometric mean ratio close to unity. In contrast, Tokra Novae MTI showed significant proportional bias relative to both comparators, indicating greater concentration-dependent deviation. Nevertheless, because none of the three methods can be regarded as a true reference method, these findings do not identify which analyzer provides the most accurate D-dimer concentration; instead, they quantify relative agreement, systematic bias, and the extent to which results may diverge in individual samples.

Collectively, these findings show that the absence of a harmonized international reference procedure for D-dimer [12,20] has direct and measurable consequences for clinical decision-making rather than being merely a theoretical limitation. This conclusion is supported by recent large-scale external proficiency data demonstrating substantial inter-assay variability around the VTE exclusion threshold and explicitly cautioning against the interchangeable use of assays [38]. Laboratories that change analyzers or operate multiple platforms in parallel cannot rely solely on manufacturer-reported performance to assume comparability. Instead, institution-specific method-comparison studies conducted according to recognized frameworks such as CLSI EP09-A3 [28] are warranted, and secondary algorithms such as age-adjusted thresholds should be revalidated for each platform rather than extrapolated from other assays [35,36,37]. The same caution applies to the use of absolute D-dimer values as semi-quantitative markers of severity, as in disseminated intravascular coagulation or COVID-19-associated coagulopathy [21,26], in which cross-platform comparisons over time or between institutions should be interpreted conservatively.

The female predominance of the cohort (62/81, 76.5%) should also be considered when interpreting the observed D-dimer distribution. Population-based data have shown higher baseline D-dimer concentrations in women than in men, suggesting that sex may contribute to interindividual variation in D-dimer levels [39]. Because the present study was designed for within-specimen comparison of three analyzers rather than for the estimation of population reference values, and each patient’s plasma was measured on all three platforms, the sex imbalance is unlikely to account for the observed pairwise analytical bias. Nevertheless, the predominance of women may limit the generalizability of the absolute concentration distribution to populations with a different sex composition.

External quality assessment (EQA) and proficiency-testing programs provide complementary evidence that D-dimer variability is not confined to the present single-center comparison. International EQA data have demonstrated substantial between-assay and between-laboratory variation, reflecting differences in antibodies, calibration, reporting units, and assay design [13,38]. These external observations support our finding that results from different D-dimer platforms should not be assumed to be numerically interchangeable. Laboratory-specific CAP proficiency-testing results were not incorporated into the present dataset because such data were not part of the prespecified patient-sample comparison; instead, published international EQA evidence is cited to place the observed inter-assay variability in context.

The continuous-scale and threshold-based analyses address complementary, rather than interchangeable, aspects of method comparability. Logarithmic transformation was used in the Bland–Altman agreement analysis because the D-dimer concentrations were right-skewed and the magnitude of between-method differences increased with concentration; back-transformation therefore allowed these differences to be expressed as geometric mean ratios. In contrast, clinical classification was intentionally based on the original measured D-dimer values, because fixed and age-adjusted decision thresholds are applied to untransformed concentrations in routine practice. Importantly, acceptable average agreement on the continuous scale does not guarantee interchangeability for an individual patient whose value lies near a decision threshold. In the present dataset, mixed classification across the three analyzers occurred in 21/81 patients (25.9%) at the fixed 500 ng/mL FEU threshold and in 18/81 patients (22.2%) at the age-adjusted thresholds. Thus, the principal clinical implication is not that one platform is inherently correct and another incorrect, but that analyzer-dependent bias may alter threshold-based classification in a clinically meaningful subset of samples. Accordingly, results from different D-dimer platforms should not be assumed to be numerically interchangeable, particularly when serial results or values close to a clinical decision limit are being interpreted.

Because all three platforms are used routinely in our laboratory, the observed inter-platform differences are directly relevant to daily practice, particularly when results obtained on different systems are compared or interpreted around clinical decision thresholds. Nevertheless, the selection and allocation of D-dimer platforms in routine laboratory practice are not determined by analytical agreement alone. Turnaround time, assay throughput, reagent and consumable costs, instrument availability, maintenance requirements, staffing and hands-on time, ease of integration into the laboratory workflow, and local test volume may all influence platform use. These operational characteristics were not measured in the present study and therefore should not be inferred from the analytical comparisons. The present findings are best used to inform local method verification and the assessment of cross-platform comparability, whereas operational or economic preferences among the three routinely used platforms require separate evaluation.

The principal strengths of this study include the simultaneous testing of identical specimens on all platforms under uniform preanalytical conditions, which minimized preanalytical variability, and the inclusion of randomly selected routine clinical specimens, which improves the applicability of the findings to everyday laboratory practice. Moreover, by jointly evaluating continuous-scale agreement (ICC, Passing–Bablok regression, and Bland–Altman analysis) and classification-level concordance at both fixed and age-adjusted cutoffs, this study addresses an aspect often left unexamined in previous comparisons [33], namely whether numerical agreement translates into concordant clinical decisions.

This study has several limitations. First, the modest sample size (*n* = 81) limits the precision of some agreement and classification estimates, as reflected by the width of several 95% confidence intervals, and reduces the reliability of subgroup or concentration-stratified analyses. Second, the single-center design may limit external generalizability, because patient spectrum, preanalytical workflow, reagent lots, calibration procedures, and analyzer-specific laboratory practices may differ across institutions. Third, imaging-confirmed VTE outcomes and standardized clinical pretest probability assessments were not available; therefore, the true diagnostic sensitivity, specificity, positive predictive value, and negative predictive value for VTE could not be estimated, and the clinical consequences of discordant classifications could not be directly verified. Fourth, VIDAS 3 served only as a comparator and not as a metrologically traceable or clinically confirmed reference method. Accordingly, the observed disagreement demonstrates non-equivalence between methods but does not establish which analyzer provides the more accurate D-dimer concentration. Fifth, repeated within-analyzer precision studies and concentration-stratified validation were not performed, which limits conclusions regarding reproducibility and the behavior of proportional bias within specific concentration ranges. In addition, owing to the absence of a metrologically traceable reference procedure, absolute measurement accuracy could not be determined; the study therefore assessed relative agreement and bias rather than analytical trueness. Consequently, the threshold-discordance analysis demonstrates potential differences in laboratory classification but cannot determine which of the discordant results would have been clinically correct for an individual patient, given the lack of an external reference method and adjudicated clinical outcomes. Finally, detailed clinical indications for D-dimer testing, comorbidities, standardized pretest probability scores, and imaging outcomes were not available in the de-identified dataset; therefore, clinical-spectrum effects and subgroup-specific diagnostic performance could not be examined.

In conclusion, although the three analyzers showed broadly acceptable agreement, they cannot be considered fully interchangeable. This has direct consequences for clinical classification, particularly for Tokra Novae MTI, which exhibited systematic proportional bias relative to both Sysmex CS-2500 and VIDAS 3 and showed lower relative sensitivity than Sysmex CS-2500 against the VIDAS 3 comparator. The strong agreement between Sysmex CS-2500 and VIDAS 3, which improved further with age adjustment, supports greater comparability between these two platforms, although complete interchangeability cannot be assumed. In contrast, the discordance observed with Tokra Novae MTI underscores the need for platform-specific validation before its results are used to guide VTE rule-out decisions. These findings indicate that the lack of international harmonization of D-dimer assays [38] has tangible implications for everyday clinical practice and support the routine performance of institution-specific method-comparison studies whenever an analyzer is selected or replaced [33]. Larger multicenter prospective studies incorporating imaging-confirmed VTE outcomes are needed to validate analyzer-specific decision thresholds and to ensure the safe cross-platform application of D-dimer-based diagnostic algorithms.

## Figures and Tables

**Figure 1:**
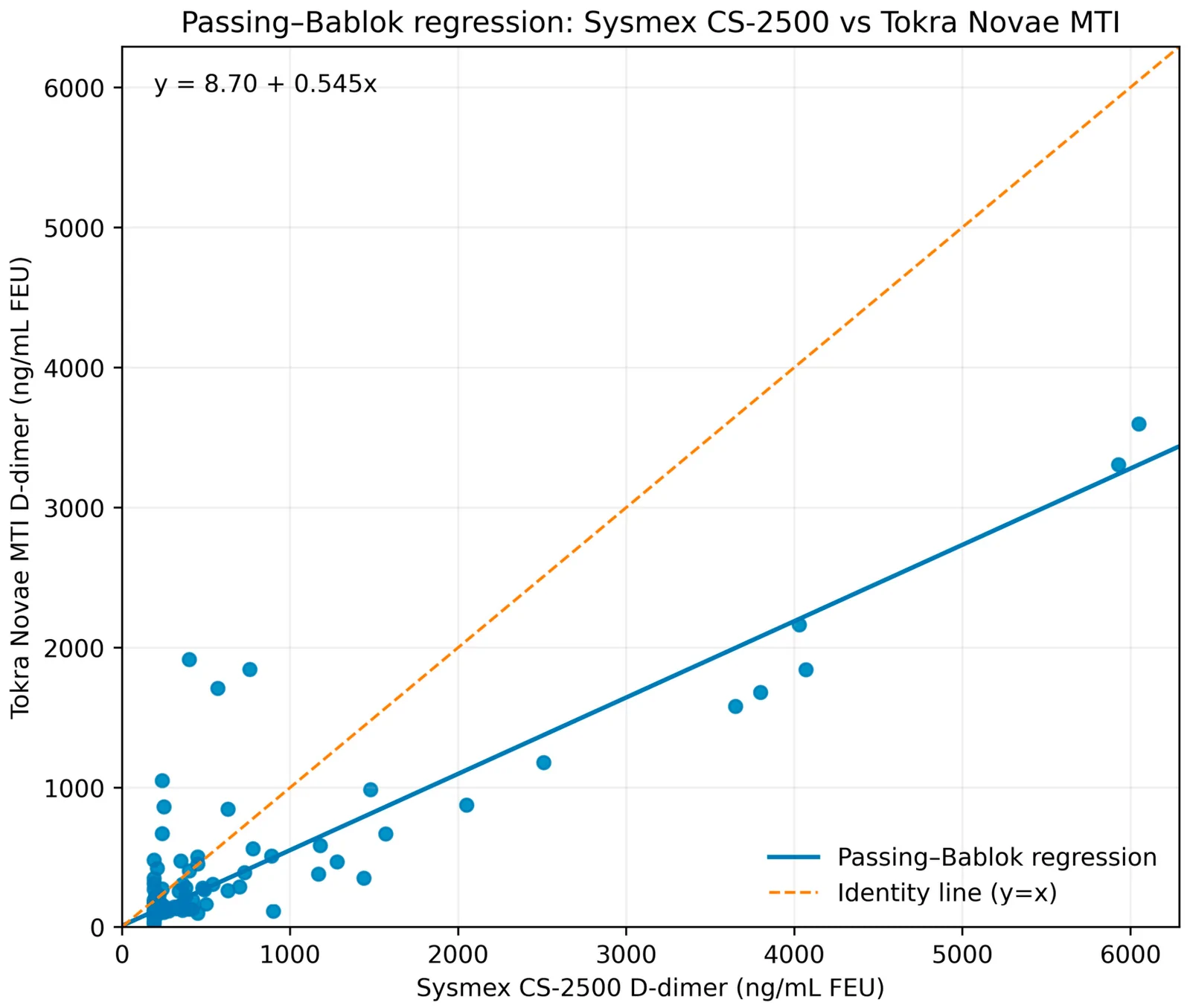
Passing–Bablok regression plot comparing Sysmex CS-2500 (X) and Tokra Novae MTI (Y) D-dimer measurements (*n* = 81). The solid line represents the Passing–Bablok regression fit, and the dashed line represents the identity line (y = x).

**Figure 2:**
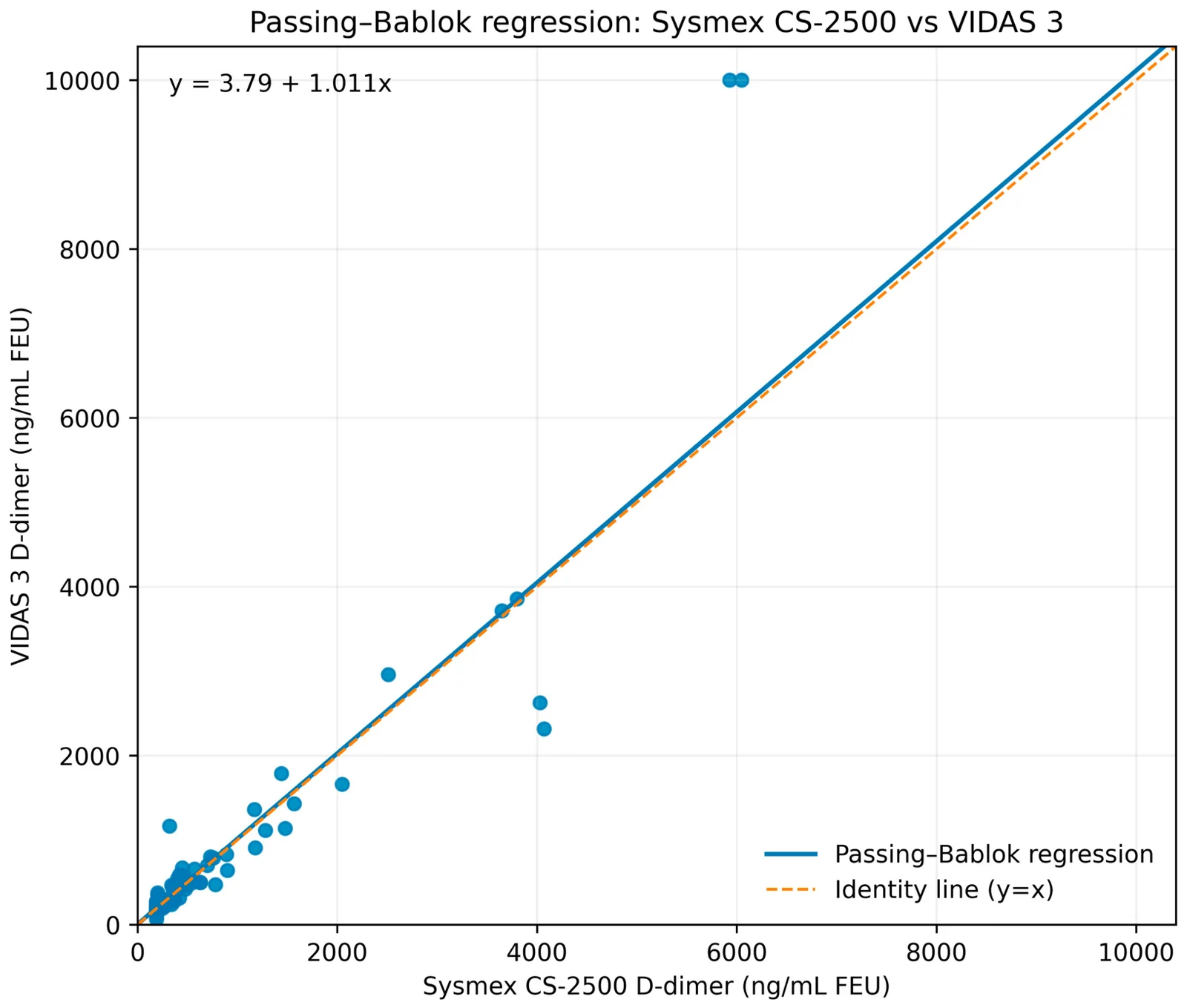
Passing–Bablok regression plot comparing Sysmex CS-2500 (X) and VIDAS 3 (Y) D-dimer measurements (*n* = 81). The solid line represents the Passing–Bablok regression fit, and the dashed line represents the identity line (y = x).

**Figure 3:**
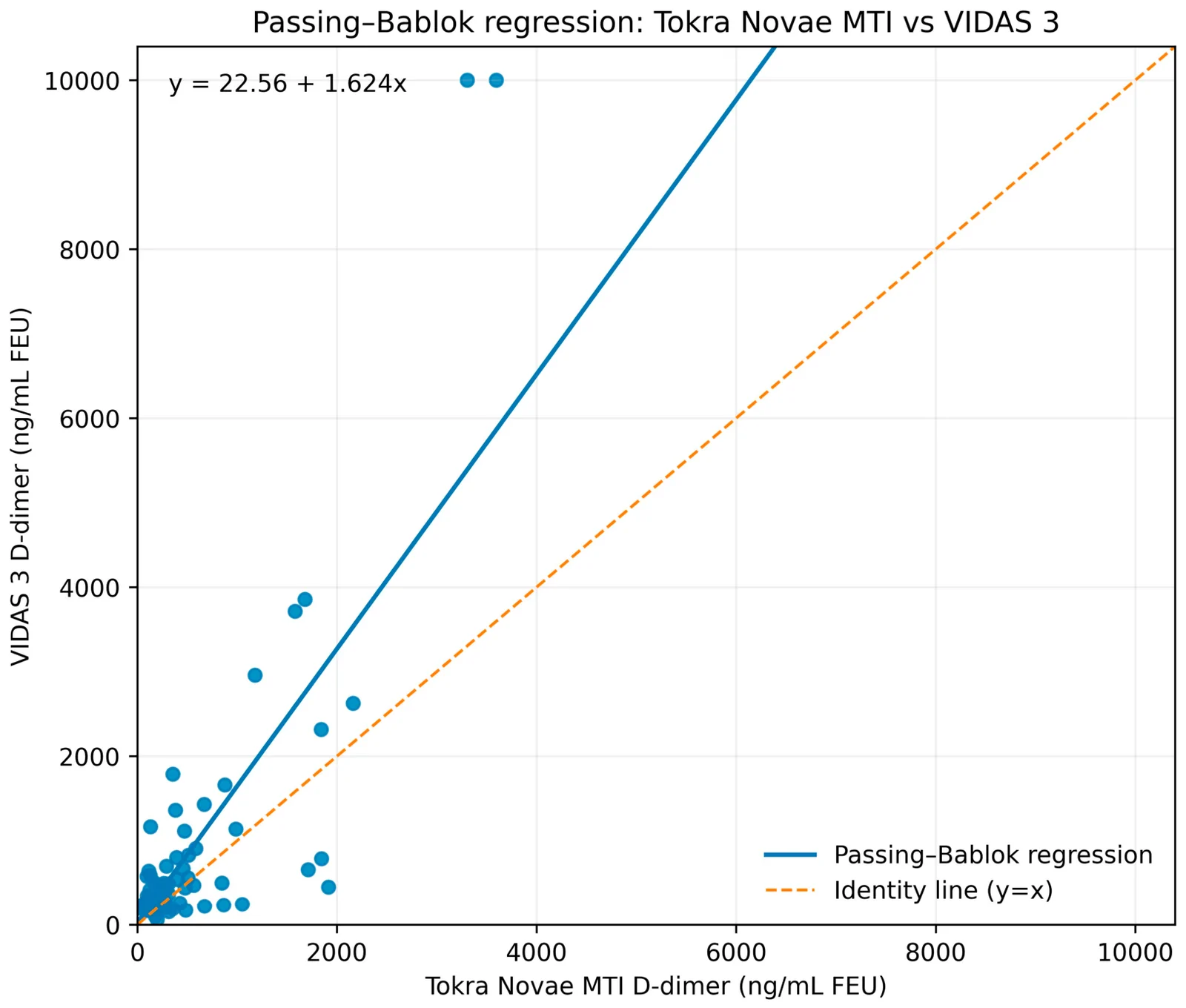
Passing–Bablok regression plot comparing Tokra Novae MTI (X) and VIDAS 3 (Y) D-dimer measurements (*n* = 81). The solid line represents the Passing–Bablok regression fit, and the dashed line represents the identity line (y = x).

**Figure 4:**
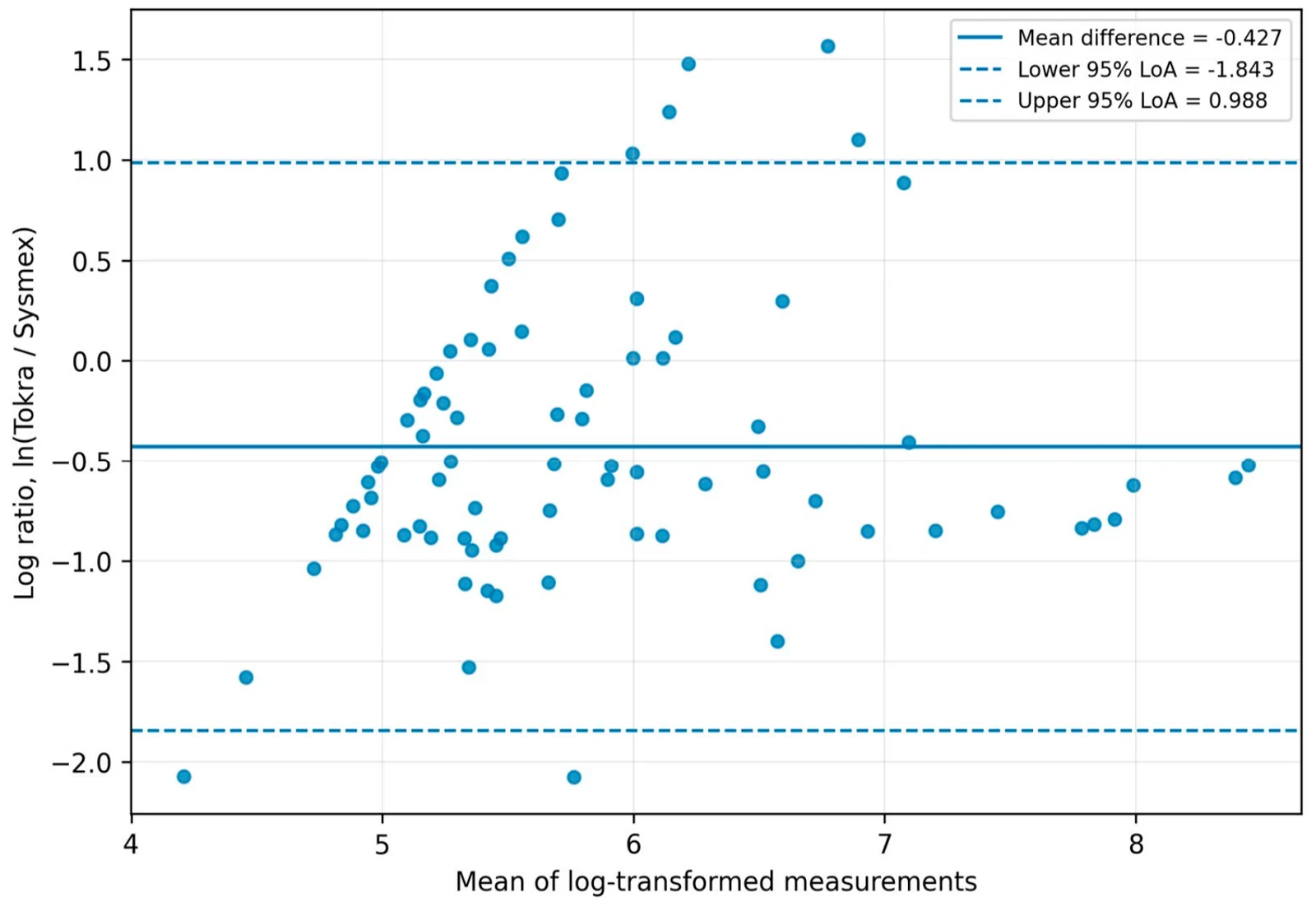
Logarithmic Bland–Altman plot of Sysmex and Tokra D-dimer measurements in the complete-case population.

**Figure 5:**
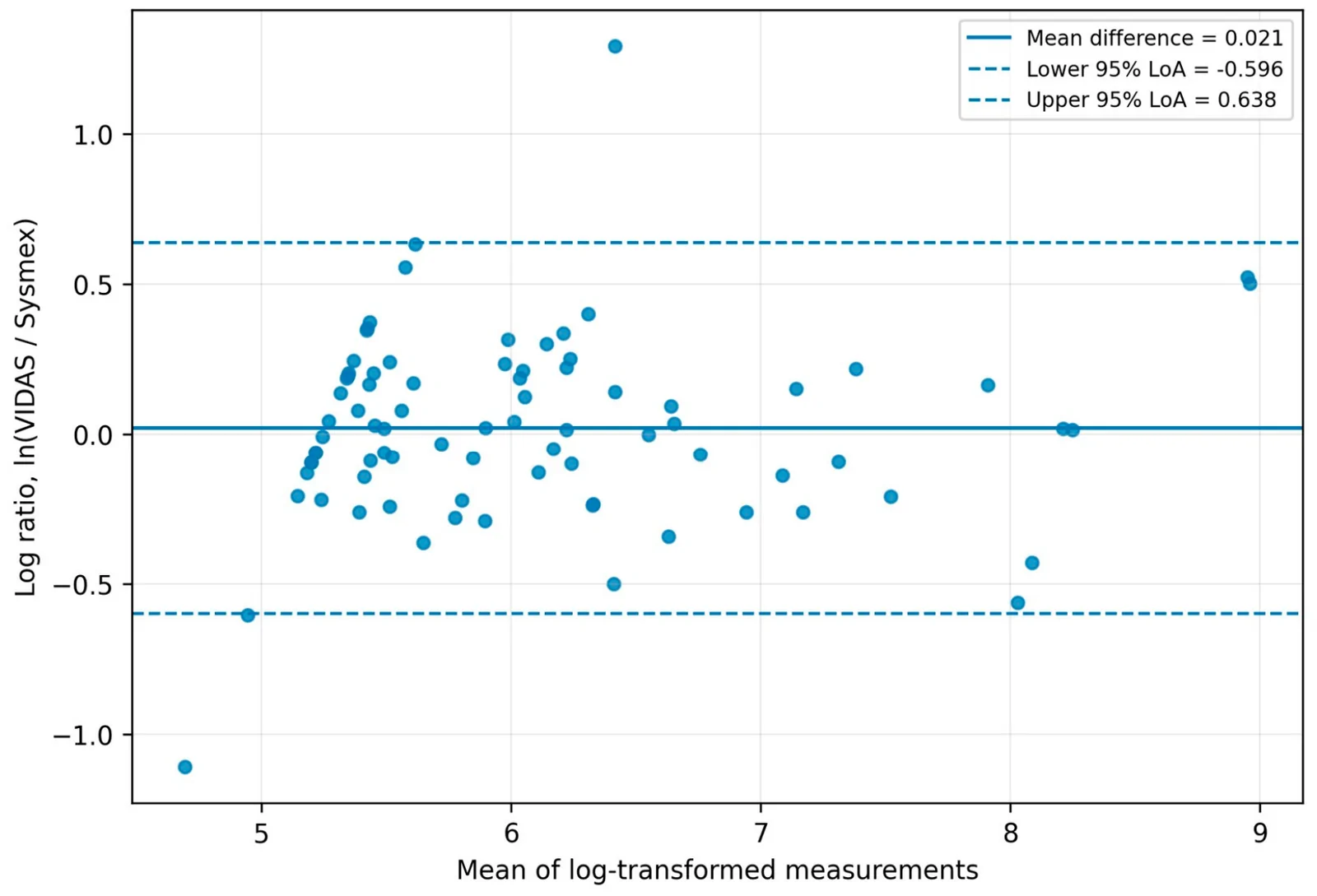
Logarithmic Bland–Altman plot of Sysmex and VIDAS D-dimer measurements in the complete-case population.

**Figure 6:**
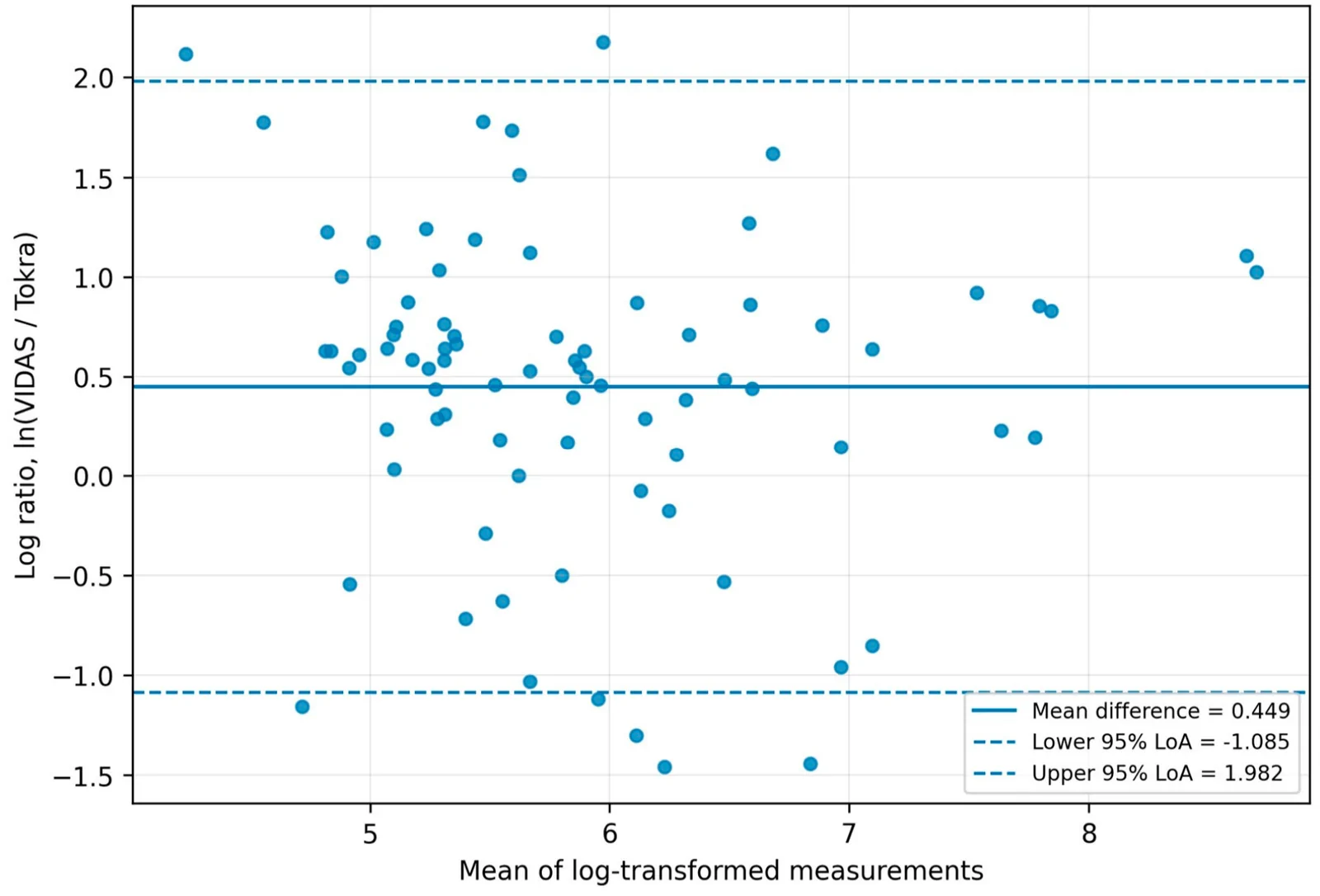
Logarithmic Bland–Altman plot of Tokra and VIDAS D-dimer measurements in the complete-case population.

**Figure 7:**
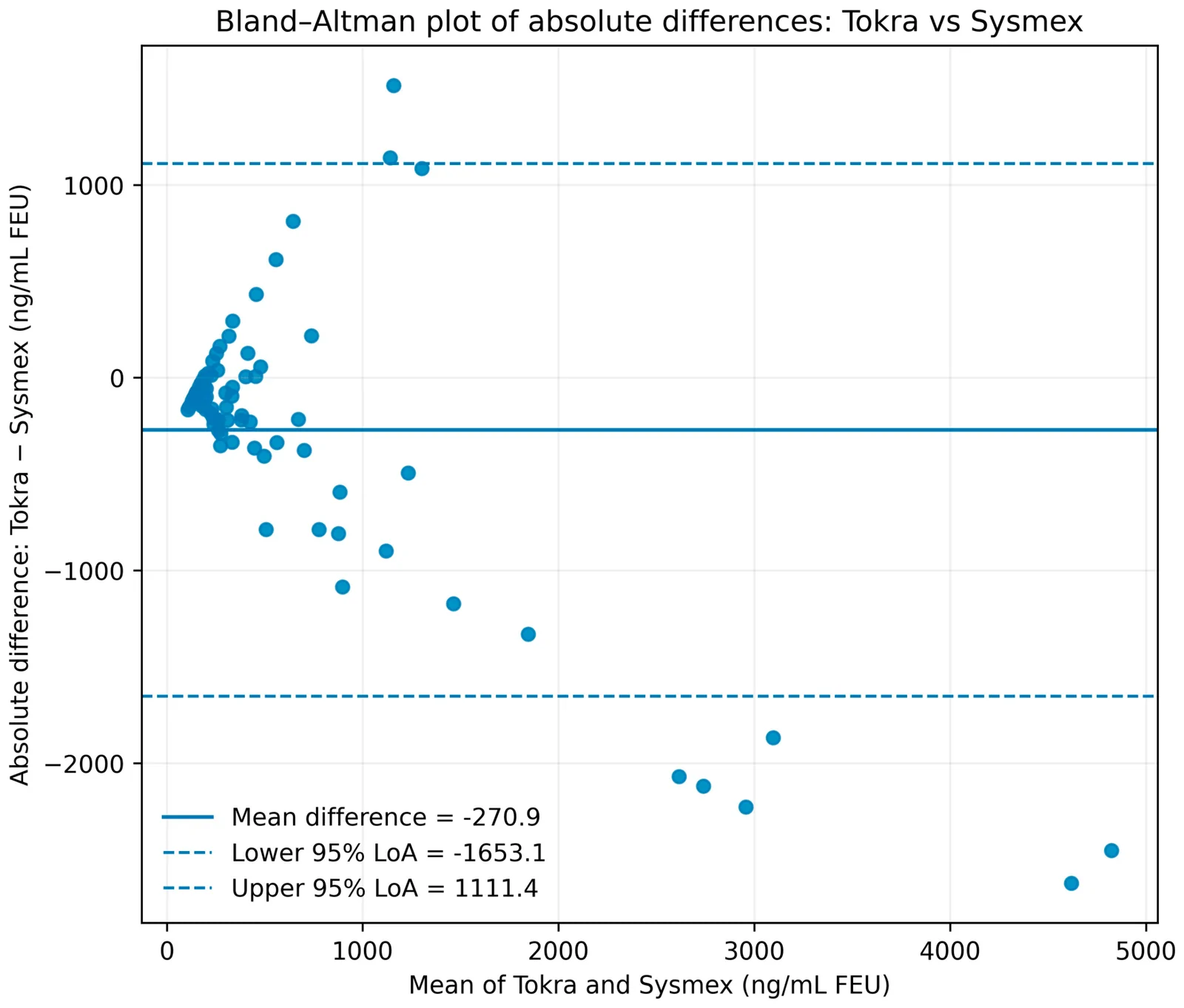
Bland–Altman plot of raw-scale differences between Tokra Novae MTI and Sysmex CS-2500 D-dimer measurements (Tokra − Sysmex) (*n* = 81). The central line indicates the mean difference, and the dashed lines indicate the 95% limits of agreement.

**Figure 8:**
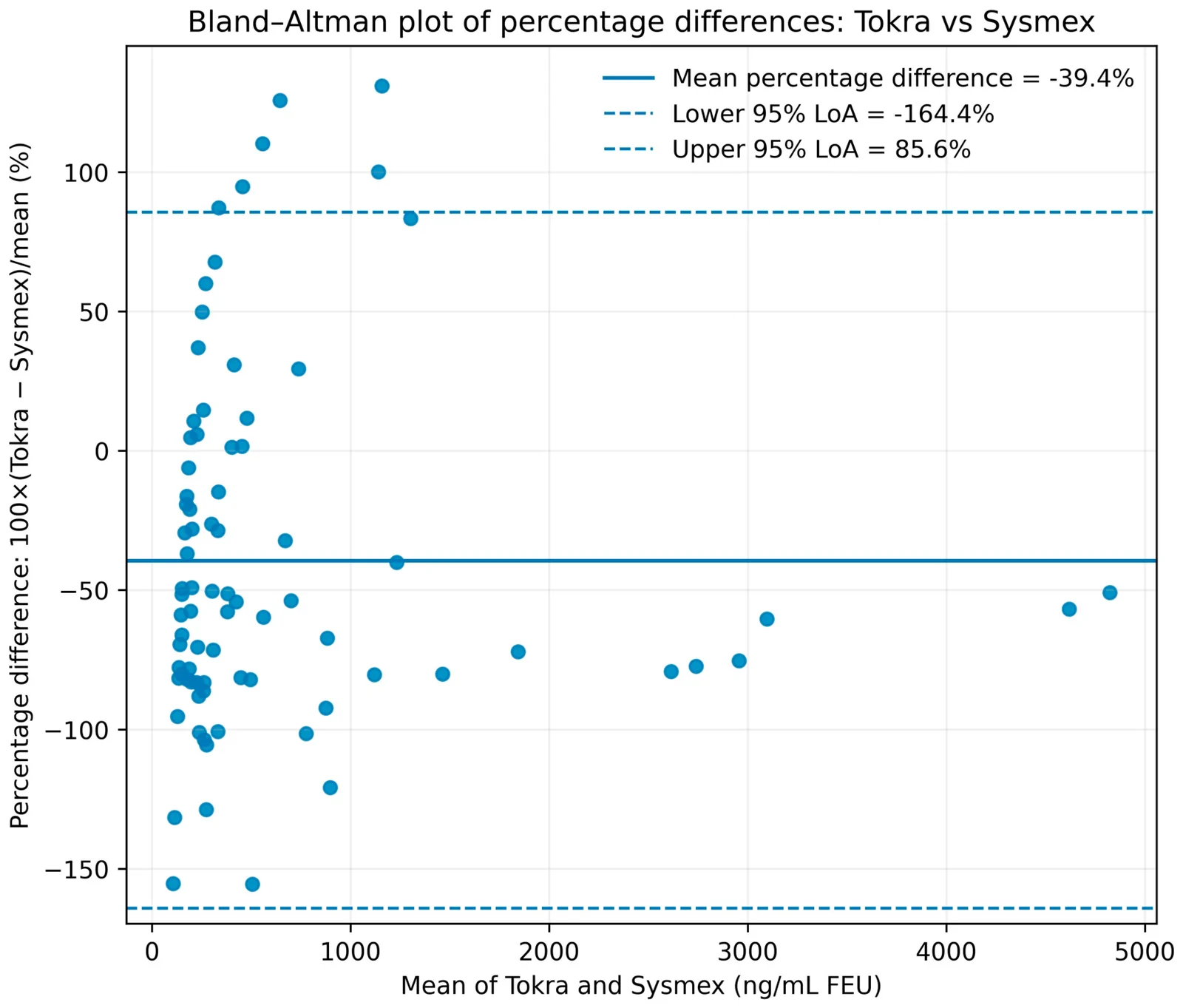
Bland–Altman plot of percentage differences between Tokra Novae MTI and Sysmex CS-2500 D-dimer measurements (*n* = 81). Percentage difference was calculated as 100 × (Tokra − Sysmex)/[(Tokra + Sysmex)/2]. The central line indicates the mean percentage difference, and the dashed lines indicate the 95% limits of agreement.

**Figure 9:**
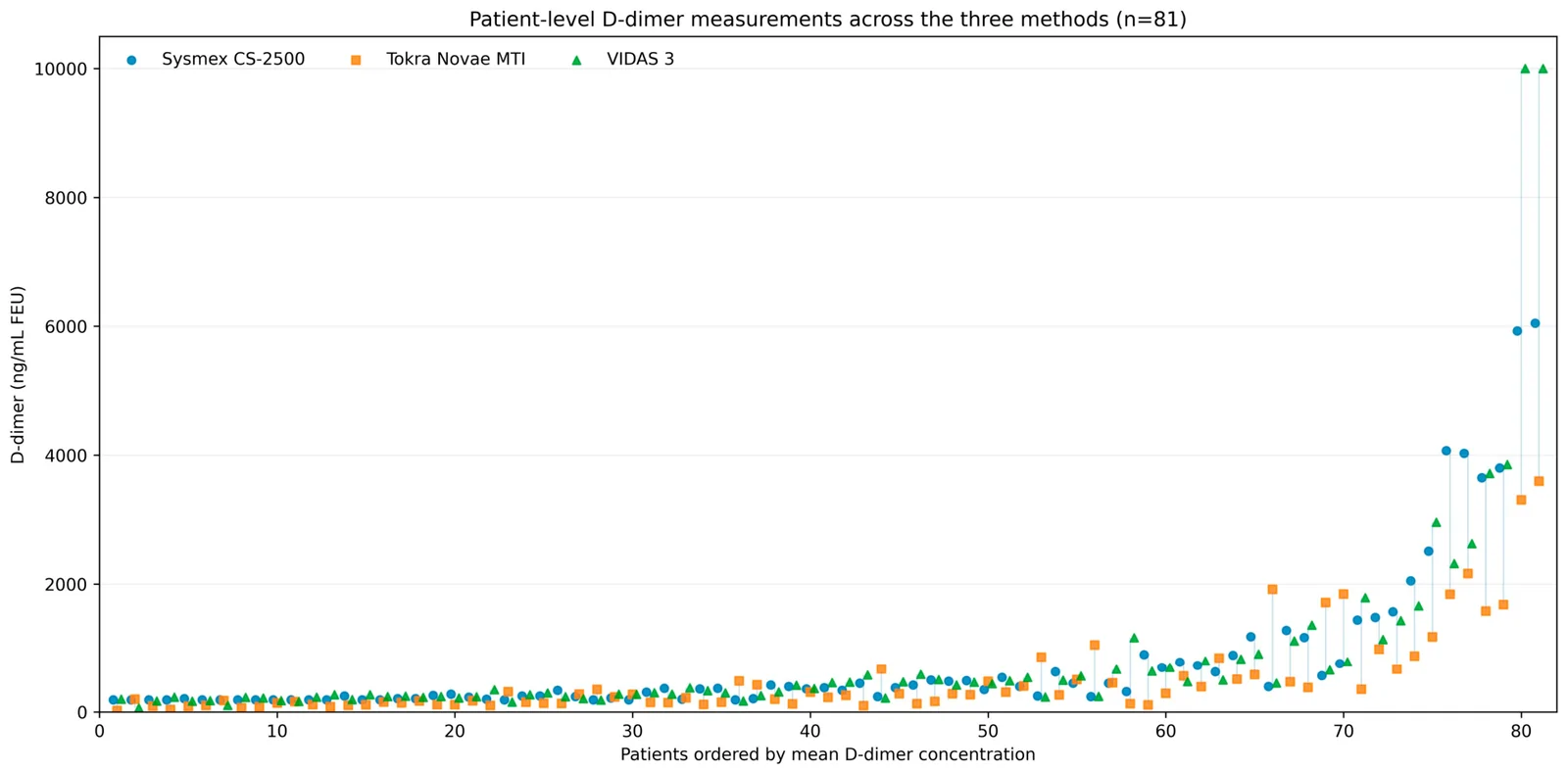
Patient-level D-dimer measurements obtained with Sysmex CS-2500, Tokra Novae MTI, and VIDAS 3 in the complete-case population (*n* = 81). Patients are ordered by their mean D-dimer concentration across the three methods; vertical segments show the within-patient range across methods.

**Table 1:** Overall absolute agreement among the three D-dimer analyzers (*n* = 81).

ICC Model	ICC	95% CI
Single measurement, absolute agreement ICC(A,1)	0.739	0.695–0.766

**Note.** A two-way mixed-effects, absolute-agreement model was used. ICC(A,1) was considered the primary estimate because a single analyzer result is used in routine clinical practice.

**Table 2:** Passing–Bablok regression results for pairwise comparisons (*n* = 81).

Comparison	Regression Equation	Intercept (95% CI)	Slope (95% CI)
Sysmex–Tokra	Tokra = 8.70 + 0.545 × Sysmex	8.70 (−30.48–49.97)	0.545 (0.450–0.645)
Sysmex–VIDAS	VIDAS = 3.79 + 1.011 × Sysmex	3.79 (−37.46–39.92)	1.011 (0.892–1.135)
Tokra–VIDAS	VIDAS = 22.56 + 1.624 × Tokra	22.56 (−83.85–97.78)	1.624 (1.236–2.188)

**Note.** The first analyzer was treated as X and the second as Y. Constant bias was inferred when the 95% CI of the intercept excluded 0, and proportional bias was inferred when the 95% CI of the slope excluded 1.

**Table 3:** Bland–Altman agreement analysis (*n* = 81).

Comparison	Geometric Mean Ratio (95% CI)	95% Ratio Limits of Agreement
Tokra/Sysmex	0.652 (0.556–0.765)	0.158–2.687
VIDAS/Sysmex	1.021 (0.953–1.095)	0.551–1.894
VIDAS/Tokra	1.566 (1.317–1.862)	0.338–7.257

**Note.** Geometric mean ratios are presented with 95% confidence intervals. A ratio close to 1 indicates similar mean measurement levels, whereas wide 95% ratio limits of agreement indicate potentially important differences at the individual-sample level.

**Table 4:** Detailed pairwise classification agreement at the fixed 500 ng/mL FEU cutoff (*n* = 81).

Comparison	Both < 500 *n* (%)	Both ≥ 500 *n* (%)	A < 500/B ≥ 500 *n* (%)	A ≥ 500/B < 500 *n* (%)	Overall Agreement, % (95% CI)	Cohen’s κ (95% CI)
Sysmex–Tokra	51 (63.0)	16 (19.8)	5 (6.2)	9 (11.1)	82.7 (73.1–89.4)	0.576 (0.380–0.773)
Sysmex–VIDAS	50 (61.7)	21 (25.9)	6 (7.4)	4 (4.9)	87.7 (78.7–93.2)	0.717 (0.554–0.880)
Tokra–VIDAS	48 (59.3)	15 (18.5)	12 (14.8)	6 (7.4)	77.8 (67.6–85.5)	0.471 (0.264–0.677)

**Note.** D-dimer values < 500 ng/mL FEU were classified as negative, and values ≥ 500 ng/mL FEU were classified as positive. A and B denote the first and second analyzers listed in the Comparison column, respectively. The four classification cells are mutually exclusive and sum to *n* = 81 for each analyzer pair.

**Table 5:** Detailed pairwise classification agreement at the age-adjusted D-dimer cutoff (*n* = 81).

Comparison	Both Below Cutoff *n* (%)	Both at/Above Cutoff *n* (%)	A Below/B at-Above *n* (%)	A at-Above/B Below *n* (%)	Overall Agreement, % (95% CI)	Cohen’s κ (95% CI)
Sysmex–Tokra	53 (65.4)	14 (17.3)	6 (7.4)	8 (9.9)	82.7 (73.1–89.4)	0.550 (0.343–0.758)
Sysmex–VIDAS	56 (69.1)	19 (23.5)	3 (3.7)	3 (3.7)	92.6 (84.8–96.6)	0.813 (0.669–0.956)
Tokra–VIDAS	52 (64.2)	13 (16.0)	9 (11.1)	7 (8.6)	80.2 (70.3–87.5)	0.486 (0.270–0.703)

**Note.** The age-adjusted cutoff was 500 ng/mL FEU for patients aged <50 years and age × 10 ng/mL FEU for patients aged ≥50 years. This age criterion was used only to calculate the patient-specific D-dimer threshold and not to define separate age groups for comparison. A and B denote the first and second analyzers listed in the Comparison column, respectively.

**Table 6:** Relative classification performance at the fixed cutoff using VIDAS as the comparator (*n* = 81).

Analyzer	Relative Sensitivity, % (95% CI)	Relative Specificity, % (95% CI)	Relative PPV, % (95% CI)	Relative NPV, % (95% CI)
Sysmex	77.8 (59.2–89.4)	92.6 (82.4–97.1)	84.0 (65.3–93.6)	89.3 (78.5–95.0)
Tokra	55.6 (37.3–72.4)	88.9 (77.8–94.8)	71.4 (50.0–86.2)	80.0 (68.2–88.2)

**Note.** VIDAS 3 was used solely as a comparator method. Values are presented with 95% confidence intervals and represent relative classification performance against VIDAS 3 rather than true diagnostic accuracy against imaging-confirmed VTE.

**Table 7:** Relative classification performance at the age-adjusted cutoff using VIDAS as the comparator (*n* = 81).

Analyzer	Relative Sensitivity, % (95% CI)	Relative Specificity, % (95% CI)	Relative PPV, % (95% CI)	Relative NPV, % (95% CI)
Sysmex	86.4 (66.7–95.3)	94.9 (86.1–98.3)	86.4 (66.7–95.3)	94.9 (86.1–98.3)
Tokra	59.1 (38.7–76.7)	88.1 (77.5–94.1)	65.0 (43.3–81.9)	85.2 (74.3–92.0)

**Note.** VIDAS 3 was used solely as a comparator method. Values are presented with 95% confidence intervals. Because imaging-confirmed VTE outcomes were not available, these estimates should not be interpreted as true rule-out performance or diagnostic accuracy.

## Data Availability

The raw data supporting the conclusions of this article will be made available by the authors on request.
